# Neurofibromatosis type 1 of the child increases birth weight

**DOI:** 10.1002/ajmg.a.61161

**Published:** 2019-04-24

**Authors:** Jussi Leppävirta, Roope A. Kallionpää, Elina Uusitalo, Tero Vahlberg, Minna Pöyhönen, Juha Peltonen, Sirkku Peltonen

**Affiliations:** ^1^ Department of Dermatology and Venereology University of Turku Turku Finland; ^2^ Department of Dermatology Turku University Hospital Turku Finland; ^3^ Department of Cell Biology and Anatomy Institute of Biomedicine, University of Turku Turku Finland; ^4^ Department of Clinical Medicine University of Turku Turku Finland; ^5^ Department of Medical and Clinical Genetics University of Helsinki Helsinki Finland; ^6^ Department of Clinical Genetics HUSLAB and Helsinki University Central Hospital Helsinki Finland

**Keywords:** anthropometrics, epidemiology, neurofibromatosis, newborn, Rasopathy

## Abstract

Neurofibromatosis type 1 (NF1) is associated with reduced adult height, but there are no cohort studies on birth size. This retrospective study includes a cohort of 1,410 persons with NF1 and a matched comparison cohort from the general population. Figures for birth size were retrieved from the administrative registers of Finland, and the data were converted to standard deviation scores (SDS), defined as standard deviation difference to the reference population. The birth weight among infants with NF1 was higher than among infants without the disorder (adjusted mean difference [95% confidence interval]: 0.53 SDS [0.19–0.87]), as was the head circumference at birth (0.58 SDS [0.26–0.90]). The birth length of the NF1 infants did not differ significantly from the comparison cohort. The birth weight in the group consisting of NF1 and non‐NF1 infants of NF1 mothers was lower than among infants of mothers in the comparison cohort (−0.28 SDS [−0.51 to −0.06]), as was the birth length (−0.22 SDS [−0.45 to 0.00]). In conclusion, the birth weight and head circumference of persons with NF1 are significantly higher than those of persons without the disorder. NF1 of the mother reduces birth weight and birth length of the infant.

## 1 INTRODUCTION

Neurofibromatosis type 1 (NF1) is a dominantly inherited cancer predisposition syndrome caused by mutations in the *NF1* gene on chromosome 17 (Gutmann et al., 2017; Jouhilahti, Peltonen, Heape, & Peltonen, 2011). The incidence of NF1 is ~1:2,000 and the prevalence 1:2,000–3,000 (Kallionpää et al., 2017; Uusitalo et al., 2015). As the *NF1* gene is prone to mutations, approximately half of the children born with NF1 have a de novo mutation (Friedman, 1999; Huson, Compston, Clark, & Harper, 1989; Poyhonen, Kytölä, & Leisti, 2000). The protein product of the *NF1* gene is neurofibromin, which acts as a tumor suppressor protein regulating the Ras signaling pathway. Hence, NF1 has been included in the group of rare genetic conditions called Rasopathies. The diagnosis of NF1 is still largely based on the National Institutes of Health (NIH) clinical criteria, which include hyperpigmented macules (*café au lait* macules), dermal neurofibromas, freckling in the axillar or inguinal regions, optic nerve gliomas, iris hamartomas (Lisch nodules), typical long‐bone abnormalities or sphenoid dysplasia, and first‐degree relative(s) with NF1 (National Institutes of Health Consensus Development Conference., 1988). Mutation analysis can confirm the diagnosis when there is a strong suspicion of the disorder but the clinical criteria are not fulfilled.

NF1 is a multi‐system disorder, and patients with NF1 have an increased risk for cancer (Uusitalo et al., 2016, 2017), skeletal fractures (Heervä et al., 2012), speech defects (Alivuotila et al., 2010), cardiovascular abnormalities (Friedman et al., 2002; Lin et al., 2000), congenital anomalies (Leppävirta et al., 2018), and learning disabilities (Krab et al., 2008). NF1 is also associated with pregnancy and delivery complications, including an increased risk for cesarean delivery, gestational hypertension, preeclampsia, preterm labor, intrauterine growth restriction (IUGR), placental abruption, and maternal cerebrovascular disease (Leppävirta et al., 2017; Terry et al., 2013).

To our knowledge, cohort studies characterizing the birth size of persons with NF1 have not been carried out. There are a few case series which suggest that the birth weight among infants born to NF1 mothers might be decreased (Segal et al., 1999; Sharma, Gulati, & Malik, 1991). This is also supported by the finding that the frequency of IUGR, commonly defined as birth weight below 10th percentile for gestational age, is increased among NF1 mothers (Leppävirta et al., 2017; Terry et al., 2013). Information on the weight of the children with NF1 is even more limited, but it has been reported that the weight of NF1 children has been similar to control subjects throughout the growth period (Clementi et al., 1999). Several studies have shown that the final height of adults with NF1 is reduced compared to the general population; the reported frequency of short stature is 7–43% (Carmi, Shohat, Metzker, & Dickerman, 1999; Clementi et al., 1999; Soucy et al., 2013; Szudek, Birch, & Friedman, 2000; Vassilopoulou‐Sellin, Klein, & Slopis, 2000). Carmi et al. (1999) observed that a short stature was more frequent than expected among persons with familial and sporadic NF1, but that growth was more severely impaired in the former group (Carmi et al., 1999). In a study involving 170 NF1 persons (where individuals were excluded if they had observed risk factors for disordered growth, such as skeletal abnormalities and precocious puberty), height was adjusted for parental height (Soucy et al., 2013). It was found that the height of persons with NF1 was decreased compared to the general population. In addition, the height of persons with NF1 was significantly decreased compared to unaffected siblings. It is known that persons with NF1 due to a *NF1* microdeletion are, on average, taller than the general population (Ning et al., 2016; Spiegel et al., 2005), but the height in early infancy is not different in comparison to persons with NF1 due to other types of mutations (Ning et al., 2016).

There are no epidemiological data available on the head circumference at birth of NF1 infants, but by adolescence, the head circumference of persons with NF1 is larger compared to controls (Szudek et al., 2000). In addition, the head circumference‐to‐height ratio is increased among children with NF1 already in early childhood. In a Finnish study, the median age when the head circumference‐to‐height exceeded the reference values by at least 2 standard deviation scores (SDS) was only 0.3 years (Karvonen et al., 2013).

Low birth weight has been associated with many perinatal complications and health issues later in life, such as increased all‐cause mortality, mortality from cardiovascular diseases, risk for childhood stunting, wheezing disorders in childhood, and coronary heart disease. High birth weight may be associated with type 1 diabetes mellitus, leukemia, and overweight in adulthood (Belbasis, Savvidou, Kanu, Evangelou, & Tzoulaki, 2016). The aim of the present register‐based total population study was to characterize the birth size of persons with NF1. In addition to what is known about the associations between birth size and health outcomes, an aim was to examine if birth size reveals new aspects of the effects of NF1 on growth.

## 2 MATERIALS AND METHODS

### 2.1 Ethical considerations

This study complies with the Declaration of Helsinki, and the study protocol was approved by the Ethics Committee of the Hospital District of Southwest Finland. Permission to run the study was obtained from the National Institute for Health and Welfare and from all secondary and tertiary referral centers in Finland.

### 2.2 Study population

The NF1‐cohort consisted of 1,410 persons with NF1, including 678 males and 732 females, acquired by searching the electronic medical registers of all secondary and tertiary referral centers of mainland Finland for inpatient and outpatient hospital visits between 1987 and 2011 accompanied with a diagnosis code for NF1. The medical records of the persons were then carefully manually reviewed to ensure that the NIH clinical criteria for NF1 were fulfilled. For the comparison cohort, 10 non‐NF1 persons, excluding first degree relatives to persons with NF1, matched for sex, age, and municipality were randomly sampled from the Population Register Centre of Finland. For 26 persons with NF1, the full number of matched persons from the general population was not acquired because of the small size of the municipality.

### 2.3 Data source

Each resident in Finland has a unique personal identity code, which is based on date of birth and sex. The code remains unchanged throughout the person's lifetime and is used to identify individuals, for example, in hospitals and medical registers. As the code remains unchanged, it can be used to follow‐up the person and cross‐link data between national registers. The Medical Birth Register of Finland is maintained by the National Institute for Health and Welfare and covers the data of all live births and of those stillbirths where the neonate has a birth weight of at least 500 g or a gestational age of at least 22 weeks. The register contains data on infants and their mothers (Gissler & Shelley, 2002; Teperi, 1993). For the current study, information from January 1, 1987 to December 31, 2013 was collected. For the infants, data on gestational age, weight, and length were available since 1987 and on head circumference since 2004. Gestational age at birth was calculated from the last menstrual period and sonography screening. Birth weight, birth length, and head circumference were measured during the first hour after delivery. For the mothers, age, parity, and smoking during pregnancy were available since 1987. Weight and height of the mother before the pregnancy and the presence of gestational diabetes were available since 2004.

The association between NF1 and birth size was studied by comparing separately the birth size of infants with NF1 with matched infants in the comparison cohort and infants of NF1 mothers with infants of matched mothers in the comparison cohort. Analyses were also carried out by stratifying the NF1‐cohort by NF1 diagnoses of the infant and the mother to form the following groups:

- NF1 infants of NF1 and non‐NF1 mothers (all NF1 infants)
- NF1 infants of non‐NF1 mothers
- NF1 infants of NF1 mothers
- NF1 and non‐NF1 infants of NF1 mothers (all infants of NF1 mothers)
- non‐NF1 infants of NF1 mothers

Often, the diagnosis of NF1 cannot be ascertained at birth but usually by age of 5 (DeBella, Szudek, & Friedman, 2000). Therefore, only infants born before January 1, 2007 were included in the analyses where the effect of the infant's NF1 was studied to ensure that the persons with NF1 were reliably included in the cohort. Because the risk for fetal and neonatal complications is increased in twin pregnancies (Cheong‐See et al., 2016), multiple pregnancies were excluded from the analysis. Birth weight, length, and head circumference were analyzed by converting the measured values into SDS, defined as the standard deviation difference in comparison to the reference population adjusted for gestational age, sex, and parity (Sankilampi, Hannila, Saari, Gissler, & Dunkel, 2013). When the information on parity was missing, only sex‐ and gestational‐age‐specific reference values were used. Birth size measures were used to classify the neonates as small for gestational age (SGA, <−2 SDS), appropriate for gestational age (AGA, ≥−2 SDS and ≤2 SDS), and large for gestational age (LGA, >2 SDS). To analyze the length‐weight‐ratio of infants, the body mass index (BMI), defined as (birth weight/birth length^2^), was calculated for each infant.

### 2.4 Statistical analysis

Continuous variables were analyzed with a linear‐mixed model and categorical variables with a generalized mixed model with binomial distribution. Random intercepts were used in the analyses to take into account case–control matching and multiple offspring. When the statistical model was not estimable with two random intercepts, only one random intercept for the study person code of the mother was used, because this had more effect on the model parameters than case–control matching. Parity was analyzed by Poisson's regression with a random intercept for the person code of the mother. Analyses were adjusted for maternal age, smoking during pregnancy, and year of the delivery, because these factors are known to have effect on birth size (Bakker et al., 2011; Jaddoe et al., 2008; Sankilampi et al., 2013). The analyses of BMI were also adjusted for parity, gestational age, and sex of the infant. Because gestational diabetes, maternal height, and maternal weight are also known to associate with birth size (Ehrenberg, Mercer, & Catalano, 2004; Griffiths, Dezateux, & Cole, 2007), additional subgroup analyses were performed and reported for infants born since 2004, as information on gestational diabetes, maternal weight, and maternal height were available only since 2004. Of note, 95% confidence intervals (CI), adjusted odds ratios (OR), and two‐tailed *p*‐values were calculated. We considered *p*‐values <0.05 statistically significant throughout the study. All the cases with missing data for outcome or confounding variables were excluded from the analysis of the corresponding outcome variable. The numbers of missing information are included in Tables 1 and 2. To avoid including birth weight, birth length, or head circumference measurements obviously entered erroneously, values >6 standard deviations above or below the reference measurement were excluded; this led to exclusion of one NF1 infant and two infants in the matched comparison cohort. In the comparison cohort, which was matched to mothers with NF1, three infants were excluded due to plausible measurement or typing errors. Personal identity codes were replaced with random study person codes to ensure anonymity. The statistical analyses were performed with the SAS statistical software, version 9.4 (SAS Institute Inc., Cary, NC).

**Table 1:** Baseline characteristics of infants with NF1 and matched infants in the comparison cohort

Characteristic	Infants with NF1 (*n* = 442)	Infants in the comparison cohort (*n* = 4,548)	*p*‐value
Maternal characteristics			
Age (years)	29.5 ± 5.4	29.0 ± 5.1	0.025
Height (cm)^a^	163.9 ± 6.6	165.6 ± 6.2	0.081
Data missing	9 (17.0)	66 (12.4)	
Weight (kg)^a^	69.0 ± 15.8	67.3 ± 15.1	0.590
Data missing	8 (15.1)	70 (13.2)	
BMI (kg/m^2^)^a^	25.4 ± 5.4	24.5 ± 5.3	0.259
Data missing	9 (17.0)	70 (13.2)	
Smoking during pregnancy			0.291
Yes	73 (16.5)	671 (14.8)	
No	359 (81.2)	3,769 (82.9)	
Data missing	10 (2.3)	108 (2.4)	
Married or cohabiting			0.116
Yes	391 (88.5)	4,139 (91.0)	
No	46 (10.4)	381 (8.4)	
Data missing	5 (1.1)	28 (0.6)	
Parity			
1+	277 (62.7)	2,740 (60.2)	0.687
0	162 (36.7)	1,786 (39.3)	
Data missing	3 (0.7)	22 (0.5)	
Gestational diabetes^a^			0.877
Yes	4 (7.5)	37 (7.0)	
No	49 (92.5)	495 (93.0)	
Data missing	0 (0.0)	0 (0.0)	
Infant characteristics			
Gestational age (weeks)	39.2 ± 1.8	39.8 ± 1.6	<0.001
Data missing	4 (0.9)	29 (0.6)	
Sex			0.809
Male	241 (54.5)	2,475 (54.4)	
Female	201 (45.5)	2,073 (45.6)	
Birth weight (g)	3,600 ± 667.0	3,573 ± 537.0	0.113
Data missing	3 (0.7)	20 (0.4)	
Birth length (cm)	49.9 ± 2.8	50.3 ± 2.3	<0.001
Data missing	4 (0.9)	28 (0.6)	
Head circumference at birth (cm)^a^	35.1 ± 2.1	34.8 ± 1.5	0.242
Data missing	7 (13.2)	62 (11.7)	
BMI (kg/m^2^)	14.3 ± 1.7	14.0 ± 1.4	<0.001
Data missing	4 (0.9)	28 (0.6)	

Data are mean ± standard deviation or *n* (%).

Abbreviation: BMI, body mass index.

a Available for the 53 infants with NF1 and 532 controls born since 2004.

**Table 2:** Baseline characteristics of mothers with NF1 and matched mothers in the comparison cohort

Characteristic	Mothers with NF1 (*n* = 357)	Mothers in the comparison cohort (*n* = 4,396)	*p*‐value
Maternal characteristics			
Age (years)	28.5 ± 5.2	28.8 ± 5.3	0.360
Height (cm)^a^	159.9 ± 5.6	165.9 ± 5.9	<0.001
Data missing	8 (5.8)	67 (3.9)	
Weight (kg)^a^	62.0 ± 12.2	67.3 ± 14.4	<0.001
Data missing	8 (5.8)	77 (4.5)	
BMI (kg/m^2^)^a^	24.2 ± 4.4	24.4 ± 4.8	0.860
Data missing	8 (5.8)	79 (4.6)	
Smoking during pregnancy			0.398
Yes	46 (12.9)	697 (15.9)	
No	293 (82.1)	3,585 (81.6)	
Data missing	18 (5.0)	114 (2.6)	
Married or cohabiting			0.200
Yes	318 (89.1)	3,964 (90.2)	
No	37 (10.4)	416 (9.5)	
Data missing	2 (0.6)	16 (0.4)	
Parity			
1+	218 (61.1)	2,516 (57.2)	0.057
0	137 (38.4)	1,867 (42.5)	
Data missing	2 (0.6)	13 (0.3)	
Gestational diabetes^a^			0.725
Yes	17 (12.2)	174 (10.2)	
No	122 (87.8)	1,530 (89.8)	
Data missing	0 (0.0)	0 (0.0)	
Infant characteristics			
Gestational age (weeks)	39.2 ± 2.4	39.8 ± 1.7	<0.001
Data missing	2 (0.6)	22 (0.5)	
Sex			0.737
Male	177 (49.6)	2,222 (50.5)	
Female	180 (50.4)	2,174 (49.5)	
Birth weight (g)	3,268 ± 695.5	3,552 ± 532.6	<0.001
Data missing	2 (0.6)	10 (0.2)	
Birth length (cm)	48.9 ± 3.3	50.3 ± 2.3	<0.001
Data missing	5 (1.4)	31 (0.7)	
Head circumference at birth (cm)^a^	34.8 ± 2.1	35.0 ± 1.6	0.095
Data missing	10 (7.2)	76 (4.5)	
BMI (kg/m^2^)	13.5 ± 1.9	14.0 ± 1.4	<0.001
Data missing	5 (1.4)	31 (0.7)	

Data are mean ± standard deviation or *n* (%).

Abbreviation: BMI, body mass index.

a Available for the 139 infants with NF1 and 1,704 controls born since 2004.

## 3 RESULTS

### 3.1 Baseline characteristics

A total of 465 NF1 infants (22 twins) born between 1987 and 2006 were identified. There were 124 infants with NF1 whose mother also had NF1. In the comparison cohort, 4,671 infants (121 twins) were identified. During the study period between 1987 and 2013, 176 females with NF1 gave birth to a total of 375 infants, including 18 twins. For the mothers in the comparison cohort, the numbers were 2,261, 4,511, and 112, respectively.

The baseline characteristics of the NF1 infants and their mothers are presented in Table 1. The mothers of NF1 infants were slightly older than the mothers of matched infants in the comparison cohort (mean: 29.5 vs. 29.0 years, *p* = 0.025), but otherwise the maternal baseline characteristics did not differ between the cohorts. The gestational age at birth of the infants with NF1 was lower than of the matched infants in the comparison cohort (39.2 vs. 39.8 weeks, *p* < 0.001), and the NF1 infants were significantly shorter (49.9 vs. 50.3 cm, *p* < 0.001) and showed higher BMI (14.3 vs. 14.0 kg/m^2^, *p* < 0.001) at birth than the matched infants in the comparison cohort.

The baseline characteristics of the mothers with NF1, their offspring, and the matched mothers in the comparison cohort are shown in Table 2. The NF1 mothers were significantly shorter (159.9 vs. 165.9 cm, *p* < 0.001*)* and weighed less (62.0 vs. 67.3 kg, *p* < 0.001) than the matched mothers in the comparison cohort, but otherwise there were no significant differences in maternal characteristics. The values for gestational age at birth (39.2 vs. 39.8 weeks, *p* < 0.001), birth length (48.9 vs. 50.3 cm*, p* < 0.001), birth weight (3,268 vs. 3,552 g*, p* < 0.001), and BMI (13.5 vs. 14.0 kg/m^2^ *, p* = <0.001) were significantly smaller among infants of NF1 mothers than among infants of the matched mothers in the comparison cohort.

### 3.2 Birth weight

Weight at birth stratified by the NF1 diagnoses of the infant and mother, described as mean SDSs, is presented in Table 3. The birth weight of the NF1 infants was statistically significantly higher than that of the infants in the comparison cohort. The mean difference in birth weight compared to comparison cohort was greater in the subgroup of NF1 infants born to non‐NF1 mothers than in the group consisting of NF1 infants of NF1 and non‐NF1 mothers. The proportion of SGA infants regarding birth weight did not differ from the comparison cohort in either group. However, the odds for being born LGA for weight were increased among NF1 infants of NF1 and non‐NF1 mothers (7.7% vs. 3.1%, adjusted OR: 2.69, 95% CI: 1.80–4.03) and among NF1 infants of non‐NF1 mothers (10.7% vs. 3.1%, adjusted OR: 3.59, 95% CI: 2.37–5.43). When only infants who were born in 2004–2006 were included and analyses were adjusted also for gestational diabetes, maternal weight and maternal height, differences in the proportion of LGA children regarding weight remained significant (NF1 infants of NF1 and non‐NF1 mothers: 9.4% vs. 1.7%, adjusted OR: 7.30, 95% CI: 1.60–33.36; NF1 infants of non‐NF1 mothers: 14.3% vs. 1.7%, adjusted OR: 9.79, 95% CI: 1.70–56.35).

**Table 3:** Birth weight of infants with NF1 and infants of NF1 mothers compared to comparison cohort

Mother/child	SDS (since 1987), adjusted mean ± SE^a^ (*n*)	Adjusted mean difference (95% CI)^a^	SDS (since 2004), adjusted mean ± SE^b^ (*n*)	Adjusted mean difference (95% CI)^b^
Comparison cohort^c^^,^ d	−0.14 ± 0.02 (4424)	Ref.	−0.09 ± 0.10 (455)	Ref.
NF1 or non‐NF1/NF1^c^	0.20 ± 0.06 (431)	0.34 (0.23 to 0.45)	0.44 ± 0.19 (43)	0.53 (0.19 to 0.87)
Comparison cohort^c^^,^ d	−0.14 ± 0.02 (4424)	Ref.	−0.07 ± 0.11^e^ (455)	Ref.
Non‐NF1/NF1^c^	0.44 ± 0.06 (306)	0.58 (0.46 to 0.71)	0.83 ± 0.25^e^ (24)	0.90 (0.46 to 1.34)^e^
Comparison cohort^c^^,^ d	−0.14 ± 0.02 (4424)	Ref.	−0.08 ± 0.10 (455)	Ref.
NF1/NF1^c^	−0.52 ± 0.11 (119)	−0.38 (−0.59 to −0.17)	−0.05 ± 0.25 (19)	0.03 (−0.47 to 0.53)
Comparison cohort^f^^,^ g	−0.14 ± 0.02 (4270)	Ref.	−0.18 ± 0.05 (1581)	Ref.
NF1/NF1 or non‐NF1^f^	−0.62 ± 0.07 (339)	−0.48 (−0.62 to −0.33)	−0.46 ± 0.11 (126)	−0.28 (−0.51 to −0.06)
Comparison cohort^c^^,^ g	−0.13 ± 0.03 (3097)	Ref.	−0.25 ± 0.10 (425)	Ref.
NF1/non‐NF1^c^	−0.82 ± 0.11 (137)	−0.70 (−0.90 to −0.49)	−1.08 ± 0.25 (24)	−0.83 (−1.29 to −0.37)

“NF1 or non‐NF1/NF1” refers to all children with NF1. “NF1/NF1 or non‐NF1” refers to all children of mothers with NF1.

Abbreviations: CI, confidence interval; SDS, standard deviation score (difference in comparison to reference population adjusted for gestational age, sexand parity); SE, standard error.

a Adjusted for maternal age, smoking during pregnancy, and year of the delivery.

b Adjusted for maternal age, gestational diabetes, smoking during pregnancy, year of the delivery, maternal weight, and maternal height.

c Infants born before 2007.

d Matched to infants with NF1.

e Only case–control matching as random variable.

f Infants born before 2014.

g Matched to mothers with NF1.

In contrast, the birth weight of the infants of mothers with NF1 was significantly decreased compared to comparison cohort. The difference was significant in the groups of NF1 and non‐NF1 infants of NF1 mothers, non‐NF1 infants of NF1 mothers, and NF1 infants of NF1 mothers. The mean difference to infants of the matched mothers in the comparison cohort was greatest in the group of non‐NF1 infants of NF1 mothers. The ORs for infant being born SGA for weight were increased among NF1 and non‐NF1 infants of NF1 mothers (10.9% vs. 2.6%, adjusted OR: 4.48, 95% CI: 2.92–6.89), non‐NF1 infants of NF1 mothers (15.3% vs. 2.7%, adjusted OR: 6.95, 95% CI: 4.06–11.91), and NF1 infants of NF1 mothers (8.9% vs. 2.5%, adjusted OR: 3.52, 95% CI: 1.66–7.48). However, when only births since 2004 were included and the analysis was adjusted for gestational diabetes, maternal height, and maternal weight, the differences in the proportion of infants born SGA lost statistical significance.

### 3.3 Birth length

The birth length of the infants with NF1 did not differ significantly from the matched infants in the comparison cohort (Table 4). Birth length among NF1 infants of non‐NF1 mothers was increased compared to infants in the comparison cohort, but when only children born between 2004 and 2006 were included and the analysis was adjusted for gestational diabetes, maternal weight, and maternal height, the difference lost statistical significance. The odds for being SGA or LGA regarding length were not statistically different among infants with NF1 compared to infants in the comparison cohort.

**Table 4:** Birth length of infants with NF1 and infants of NF1 mothers compared to comparison cohort

Mother/child	SDS (since 1987), adjusted mean ± SE^a^ (*n*)	Adjusted mean difference (95% CI)^a^	SDS (since 2004), adjusted mean ± SE^b^ (*n*)	Adjusted mean difference (95% CI)^b^
Comparison cohort^c^^,^ d	−0.16 ± 0.02 (4416)	Ref.	−0.07 ± 0.10 (453)	Ref.
NF1 or non‐NF1/NF1^c^	−0.16 ± 0.05 (430)	0.00 (−0.11 to 0.11)	0.03 ± 0.19 (43)	0.10 (−0.24 to 0.43)
Comparison cohort^c^^,^ d	−0.16 ± 0.02 (4416)	Ref.	−0.04 ± 0.11^e^ (453)	Ref.
Non‐NF1/NF1^c^	0.03 ± 0.06 (305)	0.19 (0.07 to 0.31)	0.12 ± 0.25^e^ (24)	0.16 (−0.29 to 0.61)^e^
Comparison cohort^c^^,^ d	−0.17 ± 0.02 (4416)	Ref.	−0.05 ± 0.10 (453)	Ref.
NF1/NF1^c^	−0.72 ± 0.10 (119)	−0.55 (−0.76 to −0.35)	−0.03 ± 0.27 (19)	0.02 (−0.49 to 0.53)
Comparison cohort^f^^,^ g	−0.15 ± 0.02 (4252)	Ref.	−0.20 ± 0.05 (1574)	Ref.
NF1/NF1 or non‐NF1^f^	−0.61 ± 0.07 (337)	−0.46 (−0.60 to −0.32)	−0.42 ± 0.12 (125)	−0.22 (−0.45 to 0.00)
Comparison cohort^c^^,^ g	−0.13 ± 0.03 (3086)	Ref.	−0.33 ± 0.11 (425)	Ref.
NF1/non‐NF1^c^	−0.59 ± 0.10 (136)	−0.46 (−0.66 to −0.25)	−0.81 ± 0.25 (24)	−0.48 (−0.95 to −0.01)

“NF1 or non‐NF1/NF1” refers to all children with NF1. “NF1/NF1 or non‐NF1” refers to all children of mothers with NF1.

Abbreviations: CI, confidence interval; SDS, standard deviation score (difference in comparison to reference population adjusted for gestational age, sex and parity); SE, standard error.

a Adjusted for maternal age, smoking during pregnancy, and year of the delivery.

b Adjusted for maternal age, gestational diabetes, smoking during pregnancy, year of the delivery, maternal weight, and maternal height.

c Infants born before 2007.

d Matched to infants with NF1.

e Only case–control matching as random variable.

f Infants born before 2014.

g Matched to mothers with NF1.

The infants of mothers with NF1 were shorter than infants of the matched mothers in the comparison cohort. The proportion of SGA infants regarding length was increased in the group of NF1 and non‐NF1 infants of NF1 mothers (8.8% vs. 3.3%, adjusted OR: 2.88, 95% CI: 1.85–4.51) and in the subgroup including only non‐NF1 infants of NF1 mothers (9.2% vs. 3.1%, adjusted OR: 3.38, 95% CI 1.80–6.36), when children born since 1987 were included. However, when only infants born since 2004 were included and the analysis was adjusted also for gestational diabetes, maternal weight, and maternal height, the differences in the proportion of infants born SGA lost statistical significance.

### 3.4 Head circumference

The head circumference at birth of infants with NF1 was significantly larger than among infants in the comparison cohort (Table 5). The odds for being born LGA with regard to head circumference were increased in the group including NF1 infants of NF1 and non‐NF1 mothers (8.7% vs. 2.1%, adjusted OR: 5.72, 95% CI: 1.70–19.60) and in the subgroup including NF1 infants of non‐NF1 mothers (8.0% vs. 2.1%, adjusted OR: 5.95, 95% CI: 1.09–32.41). The head circumference of infants of NF1 mothers did not significantly differ from the head circumference of infants of matched mothers in the comparison cohort.

**Table 5:** Head circumference at birth of infants with NF1 and infants of NF1 mothers compared to comparison cohort

Mother/child	SDS (since 2004), adjusted mean ± SE^a^ (*n*)	Adjusted mean difference (95% CI)^a^
Comparison cohort^b^^,^ c	−0.03 ± 0.10 (448)	Ref.
NF1 or non‐NF1/NF1^b^	0.55 ± 0.18 (42)	0.58 (0.26 to 0.90)
Comparison cohort^b^^,^ c	−0.02 ± 0.10^d^ (448)	Ref.
Non‐NF1/NF1^b^	0.52 ± 0.23^d^ (24)	0.54 (0.12 to 0.95)^d^
Comparison cohort^b^^,^ c	−0.08 ± 0.05^e^ (448)	Ref.
NF1/NF1^b^	0.36 ± 0.23^e^ (18)	0.44 (−0.02 to 0.92)^e^
Comparison cohort^f^^,^ g	−0.04 ± 0.05 (1554)	Ref.
NF1/NF1 or non‐NF1^f^	0.10 ± 0.12 (120)	0.14 (−0.09 to 0.37)
Comparison cohort^b^^,^ g	−0.19 ± 0.10 (417)	Ref.
NF1/non‐NF1^b^	−0.55 ± 0.25 (23)	−0.35 (−0.81 to 0.10)

“NF1 or non‐NF1/NF1” refers to all children with NF1. “NF1/NF1 or non‐NF1” refers to all children of mothers with NF1.

Abbreviations: CI, confidence interval; SDS, standard deviation score (difference in comparison to reference population adjusted for gestational age, sex and parity); SE, standard error.

a Adjusted for maternal age, gestational diabetes, smoking during pregnancy, year of the delivery, maternal weight, and maternal height.

b Infants born before 2007.

c Matched to infants with NF1.

d Only case–control matching as random variable.

e Only unadjusted values available due to non‐convergence of model.

f Infants born before 2014.

g Matched to mothers with NF1.

### 3.5 Body mass index

Among NF1 infants, BMI was statistically significantly higher than among infants in the comparison cohort (NF1 infants of NF1 and non‐NF1 mothers: adjusted mean 14.45 vs. 13.89, adjusted mean difference [95% CI] 0.56 [0.43–0.69]; NF1 infants of non‐NF1 mothers: 14.66 vs. 13.89, 0.77 [0.62–0.91]). When only births between 2004 and 2006 were included and analysis was adjusted for gestational diabetes, maternal weight, and maternal height, the difference remained statistically highly significant in both groups (NF1 infants of NF1 and non‐NF1 mothers: 14.83 vs. 13.91, 0.91 [0.63–1.20]; NF1 infants of non‐NF1 mothers: 15.18 vs. 13.92, 1.27 [0.74–1.79]). On the contrary, among infants born to NF1 mothers, BMI was decreased. In the group including NF1 and non‐NF1 infants of NF1 mothers, the difference was significant only among births since 1987 (13.58 vs. 13.88, −0.30 [−0.46 to −0.13]), but the difference did not reach statistical significance, when only births since 2004 were included, and analysis was adjusted additionally for gestational diabetes, maternal height, and maternal weight. In the group of non‐NF1 infants of NF1 mothers, the difference was significant among all births since 1987 (13.19 vs. 13.89, −0.71 [−0.95 to −0.47]), and among births since 2004 after adjustment of the analysis for gestational diabetes, maternal height, and maternal weight (12.99 vs. 13.90, −0.91 [−1.45 to −0.36]).

## 4 DISCUSSION

To our surprise, and probably against a general assumption, infants born with NF1 weigh more than infants without the disorder. In contrast, if the mother has NF1, the weight and length of the infant are smaller, independently of the weight and height of the mother. These conclusions are further supported by the observation that the effects were more pronounced in the subgroups where only the infant or only the mother had NF1.

No prior population‐based studies on the birth size of NF1 infants or infants of NF1 mothers have been published. In some reported case series of NF1 mothers, the birth weight of the infant has been low, which could be due to the way subjects have been recruited (Segal et al., 1999; Sharma et al., 1991). However, in a study on 247 pregnancies of 105 females with NF1, the mean birth size of neonates was no less than 3,374 g and the rate of IUGR was not increased. Importantly, however, there was no control group in that study (Dugoff & Sujansky, 1996). Epidemiological studies of pregnancies and deliveries among mothers with NF1 have reported an increased frequency of IUGR (Leppävirta et al., 2017; Terry et al., 2013), which is consistent with our findings among NF1 mothers. The reported rate of NF1 caused by microdeletion covering the *NF1* gene is 4.7–11% (Kehrer‐Sawatzki, Mautner, & Cooper, 2017). This aberration is associated with overgrowth (Ning et al., 2016; Spiegel et al., 2005). However, as accelerated growth is not evident in early childhood (Ning et al., 2016), microdeletions do not explain the increased weight of NF1 infants observed in our cohort.

In our study, the head circumference at birth was increased among infants with NF1, which is supported by the prior finding that the head circumference‐to‐height ratio is increased already in early childhood in NF1 (Karvonen et al., 2013). There are no previous data on birth length or BMI of infants with NF1 or infants of NF1 mothers.

In addition to an increased mean birth weight of NF1 infants, the proportion of NF1 infants born LGA with regard to weight was increased. LGA predisposes to multiple obstetric complications, adverse neonatal outcomes, and complications later in life (Walsh & McAuliffe, 2012). The occurrence of a head circumference >2 SDS was increased among NF1 infants, and, likewise, a large head circumference is associated with complications, for example, emergency cesarean section during labor (Elvander, Högberg, & Ekéus, 2012). These findings could also partly explain the observed increased risk for cesarean sections in NF1‐related pregnancies (Leppävirta et al., 2017; Terry et al., 2013). The infants of NF1 mothers were born SGA regarding weight significantly more often than the infants of mothers in the comparison cohort, but the result lost statistical significance when including only infants born since 2004 and adjusting for gestational diabetes, maternal weight, and maternal weight. Thus, the increased proportion of infants born SGA regarding weight could also be explained by the effects of these confounding factors. However, this is not supported by the model parameters but the loss of statistical significance is more probably caused by the decreased statistical power. Regardless of the explanation for the result finding is clinically important as SGA is known to be associated with increased risk for several short‐ and long‐term complications of the infant (Sharma, Shastri, & Sharma, 2016).

In the current study, all diagnoses of NF1 were confirmed by scrutiny of the medical records, which is a strength compared to studies where diagnoses are based only on register data. This was highlighted in our study, as a large number of persons with a diagnosis of NF1 in their medical records had to be excluded from the final study cohort because they failed to fulfill the NIH clinical criteria (Uusitalo et al., 2015). The personal identity code connected to medical registers made it possible to study the birth size of the infants retrospectively but comprehensively. This also provided an opportunity to study subgroups where the effect of the mother and the infant could be evaluated separately. In addition, the cohorts of patients with NF1 and comparison cohort were acquired independently of the Medical Birth Register which reduces any possible bias related to birth size.

A major limitation of our data is the lack of information on gestational diabetes, maternal weight, and maternal height in the pregnancies before 2004. This decreases the power of the analyses in the subgroups due to less data. However, the main results remained statistically significant also when analyzing only the births since 2004 and adjusting for gestational diabetes, maternal weight, and maternal height. Also, a lack of information on paternal height is a limitation in our study. Approximately one quarter of the fathers of the NF1 infants have NF1. The height of the father and the neonate correlate positively (Pietiläinen et al., 2001), whereas the mean adult height of the NF1 patients is reduced, and the paternal effect on the birth size of infant would, if anything, probably counteract our finding that infants with NF1 have an increased birth weight. Thus, including paternal weight and height as confounding factors in the analyses would not probably explain the finding that NF1 of the infant increases birth weight, but would further increase the statistical significance of the finding. Due to the relatively small population of Finland, the number of persons in the subgroups is limited. However, the close scrutiny of the medical records, as done in our study, to confirm the diagnosis of NF1 would be difficult with bigger study cohorts. Long‐term effects of birth size on health are known in the general population, but the subject has not been studied among patients with NF1. Thus, we do not know whether the generally known long‐term effects of birth size apply to patients with NF1.

NF1 increases brain volume leading to macrocephaly (Greenwood et al., 2005; Said et al., 1996). In our study, this is evident already among newborns, but an increased head circumference alone does not explain why NF1 infants have an increased birth weight. Increased weight at birth could be linked to altered fat and glucose metabolism or to retention of fluid during the perinatal period. Interestingly, at least two other Rasopathies, Costello and Noonan syndromes, are associated with fetal overgrowth, but the pathophysiology of the overgrowth is unclear (Smith, Podraza, & Proud, 2009). Further studies including body composition and morphometric analyses of NF1 infants at birth are needed to specify the mechanisms leading to increased weight at birth. Following the weight during the first weeks after birth would reveal if there are differences in weight loss and weight regain compared to the general population.

## 5 CONCLUSION

NF1 infants have significantly higher values for weight, head circumference, and BMI than non‐NF1 infants. In contrast, the length and weight of infants of mothers with NF1 are decreased. Both increased and decreased birth size are associated with perinatal morbidity, mortality, and health issues later in life (Belbasis et al., 2016; Boulet, Alexander, Salihu, & Pass, 2003; Morken, Klungsøyr, & Skjaerven, 2014). NF1‐related pregnancies require close monitoring during the pregnancy and the method of delivery should be assessed carefully. Because the risk for complications during the neonatal period may be increased, the neonate should be evaluated carefully.

## DATA AVAILABILITY

The Finnish National Institute for Health and Welfare provides data for researchers who meet criteria. The application for authorization can be done at https://www.thl.fi/en/web/thlfi-en/statistics/information-for-researchers/authorisation-application. Data from NF1 database may be provided for researchers who meet the criteria to access confidential data. The authors may be contacted at https://www.utu.fi/en/units/med/units/anatomy/research/Pages/peltonen.aspx.

## References

[ajmga61161-bib-0001] Alivuotila, L. , Hakokari, J. , Visnapuu, V. , Korpijaakko‐Huuhka, A.‐M. , Aaltonen, O. , Happonen, R.‐P. , … Peltonen, J. (2010). Speech characteristics in neurofibromatosis type 1. American Journal of Medical Genetics Part A, 152, 42–51. 10.1002/ajmg.a.33178 20034087

[ajmga61161-bib-0002] Bakker, R. , Steegers, E. A. P. , Biharie, A. A. , Mackenbach, J. P. , Hofman, A. , & Jaddoe, V. W. V. (2011). Explaining differences in birth outcomes in relation to maternal age: The generation R study. BJOG: An International Journal of Obstetrics & Gynaecology, 118, 500–509. 10.1111/j.1471-0528.2010.02823.x 21244614

[ajmga61161-bib-0003] Belbasis, L. , Savvidou, M. D. , Kanu, C. , Evangelou, E. , & Tzoulaki, I. (2016). Birth weight in relation to health and disease in later life: An umbrella review of systematic reviews and meta‐analyses. BMC Medicine, 14, 147 10.1186/s12916-016-0692-5 27677312 PMC5039803

[ajmga61161-bib-0004] Boulet, S. L. , Alexander, G. R. , Salihu, H. M. , & Pass, M. (2003). Macrosomic births in the United States: Determinants, outcomes, and proposed grades of risk. American Journal of Obstetrics and Gynecology, 188, 1372–1378. 10.1067/mob.2003.302 12748514

[ajmga61161-bib-0005] Carmi, D. , Shohat, M. , Metzker, A. , & Dickerman, Z. (1999). Growth, puberty, and endocrine functions in patients with sporadic or familial neurofibromatosis type 1: A longitudinal study. Pediatrics, 103, 1257–1262. 10.1542/peds.103.6.1257 10353939

[ajmga61161-bib-0006] Cheong‐See, F. , Schuit, E. , Arroyo‐Manzano, D. , Khalil, A. , Barrett, J. , Joseph, K. S. , … Thangaratinam, S. (2016). Prospective risk of stillbirth and neonatal complications in twin pregnancies: Systematic review and meta‐analysis. British Medical Journal, 354, i4353 10.1136/bmj.i4353 27599496 PMC5013231

[ajmga61161-bib-0007] Clementi, M. , Milani, S. , Mammi, I. , Boni, S. , Monciotti, C. , & Tenconi, R. (1999). Neurofibromatosis type 1 growth charts. American Journal of Medical Genetics, 87, 317–323. 10.1002/(SICI)1096-8628(19991203)87:4<317::AID-AJMG7>3.0.CO;2-X 10588837

[ajmga61161-bib-0008] DeBella, K. , Szudek, J. , & Friedman, J. M. (2000). Use of the National Institutes of Health criteria for diagnosis of neurofibromatosis 1 in children. Pediatrics, 105, 608–614. 10.1542/peds.105.3.608 10699117

[ajmga61161-bib-0009] Dugoff, L. , & Sujansky, E. (1996). Neurofibromatosis type 1 and pregnancy. American Journal of Medical Genetics, 66, 7–10. 10.1002/(SICI)1096-8628(19961202)66:1<7::AID-AJMG2>3.0.CO;2-R 8957502

[ajmga61161-bib-0010] Ehrenberg, H. M. , Mercer, B. M. , & Catalano, P. M. (2004). The influence of obesity and diabetes on the prevalence of macrosomia. American Journal of Obstetrics and Gynecology, 191, 964–968. 10.1016/j.ajog.2004.05.052 15467573

[ajmga61161-bib-0011] Elvander, C. , Högberg, U. , & Ekéus, C. (2012). The influence of fetal head circumference on labor outcome: A population‐based register study. Acta Obstetricia et Gynecologica Scandinavica, 91, 470–475. 10.1111/j.1600-0412.2012.01358.x 22229662

[ajmga61161-bib-0012] Friedman, J. M. (1999). Epidemiology of neurofibromatosis type 1. American Journal of Medical Genetics, 89, 1–6. 10.1002/(SICI)1096-8628(19990326)89:1<1::AID-AJMG3>3.0.CO;2-8 10469430

[ajmga61161-bib-0013] Friedman, J. M. , Arbiser, J. , Epstein, J. A. , Gutmann, D. H. , Huot, S. J. , Lin, A. E. , … Korf, B. R. (2002). Cardiovascular disease in neurofibromatosis 1: Report of the NF1 cardiovascular task force. Genetics in Medicine, 4, 105–111. 10.1097/00125817-200205000-00002 12180143

[ajmga61161-bib-0014] Gissler, M. , & Shelley, J. (2002). Quality of data on subsequent events in a routine medical birth register. Medical Informatics and the Internet in Medicine, 27, 33–38. 10.1080/14639230110119234 12509121

[ajmga61161-bib-0015] Greenwood, R. S. , Tupler, L. A. , Whitt, J. K. , Buu, A. , Dombeck, C. B. , Harp, A. G. , … MacFall, J. R. (2005). Brain morphometry, T2‐weighted hyperintensities, and IQ in children with neurofibromatosis type 1. Archives of Neurology, 62, 1904–1908. 10.1001/archneur.62.12.1904 16344348

[ajmga61161-bib-0016] Griffiths, L. J. , Dezateux, C. , & Cole, T. J. (2007). Differential parental weight and height contributions to offspring birthweight and weight gain in infancy. International Journal of Epidemiology, 36, 104–107. 10.1093/ije/dyl210 16984935

[ajmga61161-bib-0017] Gutmann, D. H. , Ferner, R. E. , Listernick, R. H. , Korf, B. R. , Wolters, P. L. , & Johnson, K. J. (2017). Neurofibromatosis type 1. Nature Reviews Disease Primers, 3, 17004 10.1038/nrdp.2017.4 28230061

[ajmga61161-bib-0018] Heervä, E. , Koffert, A. , Jokinen, E. , Kuorilehto, T. , Peltonen, S. , Aro, H. T. , & Peltonen, J. (2012). A controlled register‐based study of 460 neurofibromatosis 1 patients: Increased fracture risk in children and adults over 41 years of age. Journal of Bone and Mineral Research, 27, 2333–2337. 10.1002/jbmr.1685 22692994

[ajmga61161-bib-0019] Huson, S. M. , Compston, D. A. , Clark, P. , & Harper, P. S. (1989). A genetic study of von Recklinghausen neurofibromatosis in south East Wales. I. Prevalence, fitness, mutation rate, and effect of parental transmission on severity. Journal of Medical Genetics, 26, 704–711. 10.1136/jmg.26.11.704 2511318 PMC1015740

[ajmga61161-bib-0020] Jaddoe, V. W. V. , Troe, E.‐J. W. M. , Hofman, A. , Mackenbach, J. P. , Moll, H. A. , Steegers, E. A. P. , & Witteman, J. C. M. (2008). Active and passive maternal smoking during pregnancy and the risks of low birthweight and preterm birth: The generation R study. Paediatric and Perinatal Epidemiology, 22, 162–171. 10.1111/j.1365-3016.2007.00916.x 18298691

[ajmga61161-bib-0021] Jouhilahti, E.‐M. , Peltonen, S. , Heape, A. M. , & Peltonen, J. (2011). The pathoetiology of neurofibromatosis 1. The American Journal of Pathology, 178, 1932–1939. 10.1016/j.ajpath.2010.12.056 21457932 PMC3081157

[ajmga61161-bib-0022] Kallionpää, R. A. , Uusitalo, E. , Leppävirta, J. , Pöyhönen, M. , Peltonen, S. , & Peltonen, J. (2017). Prevalence of neurofibromatosis type 1 in the Finnish population. Genetics in Medicine, 20, 1082–1086. 10.1038/gim.2017.215 29215653

[ajmga61161-bib-0023] Karvonen, M. , Saari, A. , Hannila, M.‐L. , Lönnqvist, T. , Dunkel, L. , & Sankilampi, U. (2013). Elevated head circumference‐to‐height ratio is an early and frequent feature in children with neurofibromatosis type 1. Hormone Research in Pædiatrics, 79, 97–102. 10.1159/000347119 23466600

[ajmga61161-bib-0024] Kehrer‐Sawatzki, H. , Mautner, V.‐F. , & Cooper, D. N. (2017). Emerging genotype–phenotype relationships in patients with large NF1 deletions. Human Genetics, 136, 349–376. 10.1007/s00439-017-1766-y 28213670 PMC5370280

[ajmga61161-bib-0025] Krab, L. C. , Aarsen, F. K. , de Goede‐Bolder, A. , Catsman‐Berrevoets, C. E. , Arts, W. F. , Moll, H. A. , & Elgersma, Y. (2008). Impact of neurofibromatosis type 1 on school performance. Journal of Child Neurology, 23, 1002–1010. 10.1177/0883073808316366 18827266

[ajmga61161-bib-0026] Leppävirta, J. , Kallionpää, R. A. , Uusitalo, E. , Vahlberg, T. , Pöyhönen, M. , Timonen, S. , … Peltonen, S. (2017). The pregnancy in neurofibromatosis 1: A retrospective register‐based total population study. American Journal of Medical Genetics Part A, 173, 2641–2648. 10.1002/ajmg.a.38372 28815922

[ajmga61161-bib-0027] Leppävirta, J. , Kallionpää, R. A. , Uusitalo, E. , Vahlberg, T. , Pöyhönen, M. , Peltonen, J. , & Peltonen, S. (2018). Congenital anomalies in neurofibromatosis 1: A retrospective register‐based total population study. Orphanet Journal of Rare Diseases, 13, 5 10.1186/s13023-017-0756-4 29335026 PMC5769274

[ajmga61161-bib-0028] Lin, A. E. , Birch, P. H. , Korf, B. R. , Tenconi, R. , Niimura, M. , Poyhonen, M. , … Friedman, J. M. (2000). Cardiovascular malformations and other cardiovascular abnormalities in neurofibromatosis 1. American Journal of Medical Genetics, 95, 108–117. 10.1002/1096-8628(20001113)95:2<108::AID-AJMG4>3.0.CO;2-0 11078559

[ajmga61161-bib-0029] Morken, N.‐H. , Klungsøyr, K. , & Skjaerven, R. (2014). Perinatal mortality by gestational week and size at birth in singleton pregnancies at and beyond term: A nationwide population‐based cohort study. BMC Pregnancy and Childbirth, 14, 172 10.1186/1471-2393-14-172 24885576 PMC4037279

[ajmga61161-bib-0030] National Institutes of Health Consensus Development Conference . (1988). Neurofibromatosis conference statement. National Institutes of Health consensus development conference. Archives of Neurology, 45, 575–578. 10.1001/archneur.1988.00520290115023 3128965

[ajmga61161-bib-0031] Ning, X. , Farschtschi, S. , Jones, A. , Kehrer‐Sawatzki, H. , Mautner, V.‐F. , & Friedman, J. M. (2016). Growth in neurofibromatosis 1 microdeletion patients. Clinical Genetics, 89, 351–354. 10.1111/cge.12632 26111455

[ajmga61161-bib-0032] Pietiläinen, K. H. , Kaprio, J. , Räsänen, M. , Winter, T. , Rissanen, A. , & Rose, R. J. (2001). Tracking of body size from birth to late adolescence: Contributions of birth length, birth weight, duration of gestation, parents' body size, and twinship. American Journal of Epidemiology, 154, 21–29. 10.1093/aje/154.1.21 11427401

[ajmga61161-bib-0033] Poyhonen, M. , Kytölä, S. , & Leisti, J. (2000). Epidemiology of neurofibromatosis type 1 (NF1) in northern Finland. Journal of Medical Genetics, 37, 632–636. 10.1136/jmg.37.8.632 10991696 PMC1734645

[ajmga61161-bib-0034] Said, S. M. A. , Yeh, T.‐L. , Greenwood, R. S. , Whitt, J. K. , Tupler, L. A. , & Krishnan, K. R. R. (1996). MRI morphometric analysis and neuropsychological function in patients with neurofibromatosis. Neuroreport, 7, 1941–1944. 10.1097/00001756-199608120-00015 8905698

[ajmga61161-bib-0035] Sankilampi, U. , Hannila, M.‐L. , Saari, A. , Gissler, M. , & Dunkel, L. (2013). New population‐based references for birth weight, length, and head circumference in singletons and twins from 23 to 43 gestation weeks. Annals of Medicine, 45, 446–454. 10.3109/07853890.2013.803739 23768051

[ajmga61161-bib-0036] Segal, D. , Holcberg, G. , Sapir, O. , Sheiner, E. , Mazor, M. , & Katz, M. (1999). Neurofibromatosis in pregnancy: Maternal and perinatal outcome. European Journal of Obstetrics & Gynecology and Reproductive Biology, 84, 59–61. 10.1016/S0301-2115(98)00255-3 10413228

[ajmga61161-bib-0037] Sharma, J. B. , Gulati, N. , & Malik, S. (1991). Maternal and perinatal complications in neurofibromatosis during pregnancy. International Journal of Gynecology & Obstetrics, 34, 221–227. 10.1016/0020-7292(91)90353-7 1673938

[ajmga61161-bib-0038] Sharma, D. , Shastri, S. , & Sharma, P. (2016). Intrauterine growth restriction: Antenatal and postnatal aspects. Clinical Medicine Insights: Pediatrics, 10, 67–83. 10.4137/CMPed.S40070 27441006 PMC4946587

[ajmga61161-bib-0039] Smith, L. P. , Podraza, J. , & Proud, V. K. (2009). Polyhydramnios, fetal overgrowth, and macrocephaly: Prenatal ultrasound findings of Costello syndrome. American Journal of Medical Genetics Part A, 149, 779–784. 10.1002/ajmg.a.32778 19288554

[ajmga61161-bib-0040] Soucy, E. A. , van Oppen, D. , Nejedly, N. L. , Gao, F. , Gutmann, D. H. , & Hollander, A. S. (2013). Height assessments in children with neurofibromatosis type 1. Journal of Child Neurology, 28, 303–307. 10.1177/0883073812446310 22752476 PMC3947790

[ajmga61161-bib-0041] Spiegel, M. , Oexle, K. , Horn, D. , Windt, E. , Buske, A. , Albrecht, B. , … Tinschert, S. (2005). Childhood overgrowth in patients with common NF1 microdeletions. European Journal of Human Genetics, 13, 883–888. 10.1038/sj.ejhg.5201419 15856072

[ajmga61161-bib-0042] Szudek, J. , Birch, P. , & Friedman, J. M. (2000). Growth in North American white children with neurofibromatosis 1 (NF1). Journal of Medical Genetics, 37, 933–938. 10.1136/jmg.37.12.933 11106357 PMC1734506

[ajmga61161-bib-0043] Teperi, J. (1993). Multi method approach to the assessment of data quality in the Finnish medical birth registry. Journal of Epidemiology & Community Health, 47, 242–247. 10.1136/jech.47.3.242 8350040 PMC1059775

[ajmga61161-bib-0044] Terry, A. R. , Barker, F. G. , Leffert, L. , Bateman, B. T. , Souter, I. , & Plotkin, S. R. (2013). Neurofibromatosis type 1 and pregnancy complications: A population‐based study. American Journal of Obstetrics and Gynecology, 209, 46.e1–46.e8. 10.1016/j.ajog.2013.03.029 23535241

[ajmga61161-bib-0045] Uusitalo, E. , Leppävirta, J. , Koffert, A. , Suominen, S. , Vahtera, J. , Vahlberg, T. , … Peltonen, S. (2015). Incidence and mortality of neurofibromatosis: A total population study in Finland. Journal of Investigative Dermatology, 135, 904–906. 10.1038/jid.2014.465 25354145

[ajmga61161-bib-0046] Uusitalo, E. , Rantanen, M. , Kallionpää, R. A. , Pöyhönen, M. , Leppävirta, J. , Ylä‐Outinen, H. , … Peltonen, J. (2016). Distinctive cancer associations in patients with neurofibromatosis type 1. Journal of Clinical Oncology, 34, 1978–1986. 10.1200/JCO.2015.65.3576 26926675

[ajmga61161-bib-0047] Uusitalo, E. , Kallionpää, R. A. , Kurki, S. , Rantanen, M. , Pitkäniemi, J. , Kronqvist, P. , … Peltonen, J. (2017). Breast cancer in neurofibromatosis type 1: Overrepresentation of unfavourable prognostic factors. British Journal of Cancer, 116, 211–217. 10.1038/bjc.2016.403 27931045 PMC5243991

[ajmga61161-bib-0048] Vassilopoulou‐Sellin, R. , Klein, M. J. , & Slopis, J. K. (2000). Growth hormone deficiency in children with neurofibromatosis type 1 without suprasellar lesions. Pediatric Neurology, 22, 355–358. 10.1016/S0887-8994(00)00123-5 10913726

[ajmga61161-bib-0049] Walsh, J. M. , & McAuliffe, F. M. (2012). Prediction and prevention of the macrosomic fetus. European Journal of Obstetrics & Gynecology and Reproductive Biology, 162, 125–130. 10.1016/j.ejogrb.2012.03.005 22459652

